# AAV-mediated upregulation of VDAC1 rescues the mitochondrial respiration and sirtuins expression in a SOD1 mouse model of inherited ALS

**DOI:** 10.1038/s41420-024-01949-w

**Published:** 2024-04-16

**Authors:** Andrea Magrì, Cristiana Lucia Rita Lipari, Antonella Caccamo, Giuseppe Battiato, Stefano Conti Nibali, Vito De Pinto, Francesca Guarino, Angela Messina

**Affiliations:** 1 Department of Biological, Geological and Environmental Sciences, University of Catania, Via S. Sofia 97, 95123 Catania, Italy; 2 we.MitoBiotech s.r.l., C.so Italia 172, 95125 Catania, Italy; 3 Department of Biomedical and Biotechnological Sciences, University of Catania, Via S. Sofia 97, 95123 Catania, Italy; 4 Department of Chemical, Biological, Pharmaceutical and Environmental Sciences, University of Messina, V.le F. Stagno d’Alcontres 32, 98166 Messina, Italy

**Keywords:** Molecular neuroscience, Porins

## Abstract

Mitochondrial dysfunction represents one of the most common molecular hallmarks of both sporadic and familial forms of amyotrophic lateral sclerosis (ALS), a neurodegenerative disorder caused by the selective degeneration and death of motor neurons. The accumulation of misfolded proteins on and within mitochondria, as observed for SOD1 G93A mutant, correlates with a drastic reduction of mitochondrial respiration and the inhibition of metabolites exchanges, including ADP/ATP and NAD^+^/NADH, across the Voltage-Dependent Anion-selective Channel 1 (VDAC1), the most abundant channel protein of the outer mitochondrial membrane. Here, we show that the AAV-mediated upregulation of VDAC1 in the spinal cord of transgenic mice expressing SOD1 G93A completely rescues the mitochondrial respiratory profile. This correlates with the increased activity and levels of key regulators of mitochondrial functions and maintenance, namely the respiratory chain Complex I and the sirtuins (Sirt), especially Sirt3. Furthermore, the selective increase of these mitochondrial proteins is associated with an increase in Tom20 levels, the receptor subunit of the TOM complex. Overall, our results indicate that the overexpression of VDAC1 has beneficial effects on ALS-affected tissue by stabilizing the Complex I-Sirt3 axis.

## Introduction

Amyotrophic lateral sclerosis (ALS) is the most common motor neuron disease, characterized by the degeneration of upper motor neurons in the motor cortex and lower motor neurons in the brainstem and spinal cord. ALS progression causes the denervation of skeletal muscles leading to atrophy, paralysis, and death from respiratory failure within a few years of the disease onset [1, 2]. ALS is a heterogeneous and multifactorial disorder with a predominantly sporadic onset, albeit a family history is present in ~10% of cases, with an autosomal Mendelian dominant pattern of inheritance [3, 4]. More than 30 genes are causative or contribute to ALS, and approximately one-fifth of cases with genetic origin depend on mutations in the gene encoding Cu/Zn Superoxide Dismutase (SOD1), a ubiquitously expressed cytosolic antioxidant enzyme [5–7]. Although the precise pathological mechanism linking SOD1 mutants to motor neurons degeneration is still debated, it is widely accepted that ALS-linked mutations trigger the adoption of misfolded conformations that lead to an aberrant hydrophobic behavior and protein accumulation within cells [8–11]. Interestingly, this accumulation correlates with the selective recruitment of misfolded SOD1 to the cytosolic surface of mitochondria exclusively in the affected tissues [12–14].

Mitochondria are pivotal for motor neurons maintenance and survival, being the primary source of ATP in this high-energy demand cell type. The accumulation of SOD1 mutants at the organelle level, as observed for the dismutase-active SOD1 G93A mutant, is associated with the appearance of mitochondrial structural abnormalities and fragmentation, reduced import of mitochondrial proteins into the organelle, inhibition of respiratory complexes activity and ATP production, and impairment of mitophagy [15–21]. Therefore, it is not surprising that mitochondrial dysfunction is considered a crucial event in the degeneration of motor neurons in ALS.

The Voltage-Dependent Anion-selective Channel (VDAC), also known as mitochondrial porin, is a conserved family of outer mitochondrial membrane (OMM) channel proteins ubiquitously expressed from yeast to mammals [22–24]. Among the three human isoforms, VDAC1 is the most abundant and allows for the passive diffusion of small hydrophilic molecules across the OMM, which includes ATP/ADP, NAD^+^/NADH, Krebs’ cycle intermediates and ions (Mg^2+^, Ca^2+^, Na^+^, Cl^−^) [25–27]. Moreover, being the mitochondrial docking site for glycolytic Hexokinases (HKs) enzymes, VDAC1 participates in the regulation of the whole cell metabolism and apoptosis [28–31].

As previously reported, the expression of VDAC1 does not appear to be altered in ALS models stably expressing SOD1 G93A, such as the motor neuronal cell line NSC-34, transgenic mice and rats [19, 32, 33]. At the same time, VDAC1 directly mediates the mitochondrial toxicity of SOD1 mutants in ALS motor neurons, being the main interactor of misfolded proteins at the organelle in the affected tissues [33]. Precisely, the interaction between VDAC1 and SOD1 G93A dramatically decreases the channel conductance and ADP accumulation within the mitochondrion and interferes with the physiological interaction of VDAC1 with HKs [33–35]. As a result, the downregulation of VDAC1 expression in SOD1 transgenic rats accelerates the disease onset and further reduces animals’ survival [33].

On these premises, we investigated whether the upregulation of VDAC1, following a neonatal intraspinal injection of an adeno-associated virus (AAV), could counteract the mitochondrial dysfunction typical of ALS. Here, we found that stably enhancing the expression of VDAC1 in the spinal cord of pre-symptomatic SOD1 G93A transgenic mice restores the mitochondrial respiratory profile to that of wild-type mice. This process appears to be the result of a selective increase in the function and/or expression of key mitochondrial proteins: Complex I, a component of the respiratory chain, specific members of the mitochondrial sirtuins (Sirt), a family of deacetylases involved in the regulation of many mitochondrial pathways, and the receptor subunit of the Translocase of the Outer Membrane (TOM) complex, Tom20, the most important gateway for the entry of mitochondrial pre-proteins within the organelle. Together, these data indicate that the upregulation of VDAC1 might be a valid treatment for ALS.

## Results

### AAV2/5 vector efficiently allows the long-term upregulation of VDAC1 in the spinal cord of transgenic ALS mice

For this study, we used the commercially available mouse strain B6.Cg-Tg(SOD1*G93A)1Gur/J, herein simply referred to as transgenic mice, and the related control strain C57BL6/J, herein simply referred to as wild-type. As reported by literature, this transgenic strain develops hindlimb and non-hindlimb tremors typical of ALS with a mean onset of about 100 days, which corresponds to approximately 14 weeks of age [36]. Moreover, as demonstrated by our previous report, transgenic mice of this genetic background show high expression level of SOD1 G93A in the spinal cord, which is associated with a dramatic decrease in the whole respiratory profile of mitochondria [19].

**Fig. 1:**
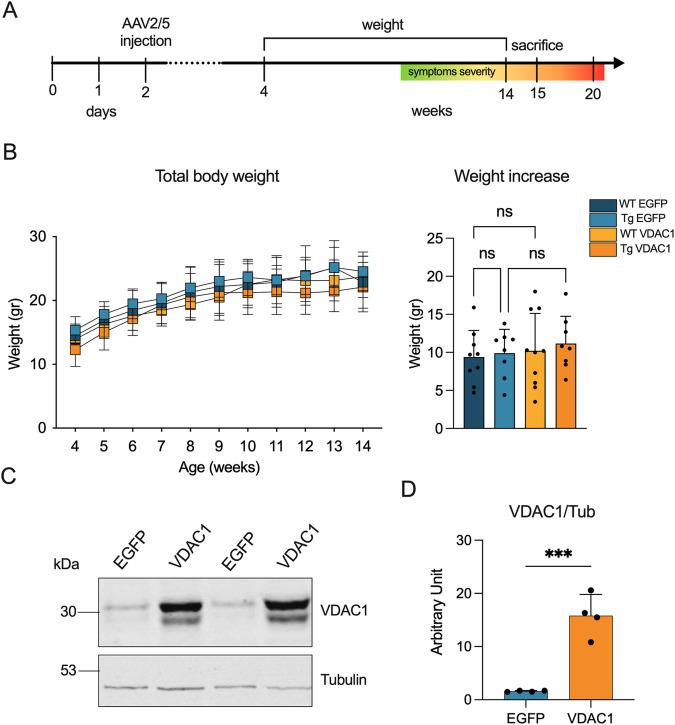
AAV2/5-mediated intraspinal injection of VDAC1 gene efficiently allows the overexpression of the relative protein. **A** Schematization of the experimental plan followed in this work. To obtain four different experimental groups, AAV2/5 constructs, carrying VDAC1 or EGFP sequence, were delivered to wild-type and transgenic mice by intraspinal injection at P2. Then, body weight was monitored and mice were sacrificed at week 15, in concomitance with the occurrence of motor impairment. **B** Analysis of body weight from week 4 to 14 and body weight increase. Data are expressed as median or means ± SEM of *n* = 8 independent measurements per experimental group, and statistically analyzed by one-way ANOVA; *ns*, not significant. **C** Representative Western blots of spinal cord total homogenates extracted from transgenic mice previously injected with VDAC1 or EGFP construct aimed at investigating VDAC1 expression. Tubulin was used as loading control. **D** Relative quantification of VDAC1 expression of Western blot in (**C**). Data are expressed as mean ± SEM of *n* = 4 independent measurements and statistically analyzed by unpaired Student *t*-test, with ***p < 0.001.

Figure 1A shows the timeline used for this study. To assess the potential use of VDAC1 overexpression as therapeutical tool in this pathological context, we used an AAV vector of serotype 2 packaged in the capsid of serotype 5. Among the various serotypes, the injection of an AAV2/5 vector in the spinal cord of ALS mice is the most efficient route for the widespread transduction of the carried gene [37]. Briefly, AAV2/5 constructs carrying the VDAC1 gene or EGFP were delivered at post-natal day 2 (P2) at the lumbar region of the spinal cord of wild-type and transgenic mice. Notably, the efficiency of the gene delivery was previously validated by analyzing the expression of EGFP by Western blot in a group of wild-type mice injected with the AAV2/5-EGFP construct (see Supplementary Fig. 1).

After the injection, mice were monitored and their body weight was checked every week to exclude any toxic effect linked to VDAC1 overexpression. No significant difference in body weight was noticed between wild-type and transgenic mice either expressing VDAC1 or EGFP (Fig. 1B). At 15 weeks post injection, the mice were sacrificed and the upregulation of the VDAC1 gene was then investigated by Western blot on spinal cord total lysates from transgenic mice. As shown in Fig. 1C (and in the full scan of Supplementary Fig. 2), mice injected with the AAV2/5 construct carrying the VDAC1 sequence showed a robust, long-term stable expression of the respective protein. From the comparison with mice injected with the EGFP construct, VDAC1 band intensity was about 10-fold higher in comparison to the endogenous one (*p* < 0.001, *n* = 4, Fig. 1D).

### Overexpression of VDAC1 rescues mitochondrial respiration in transgenic mice

**Fig. 2:**
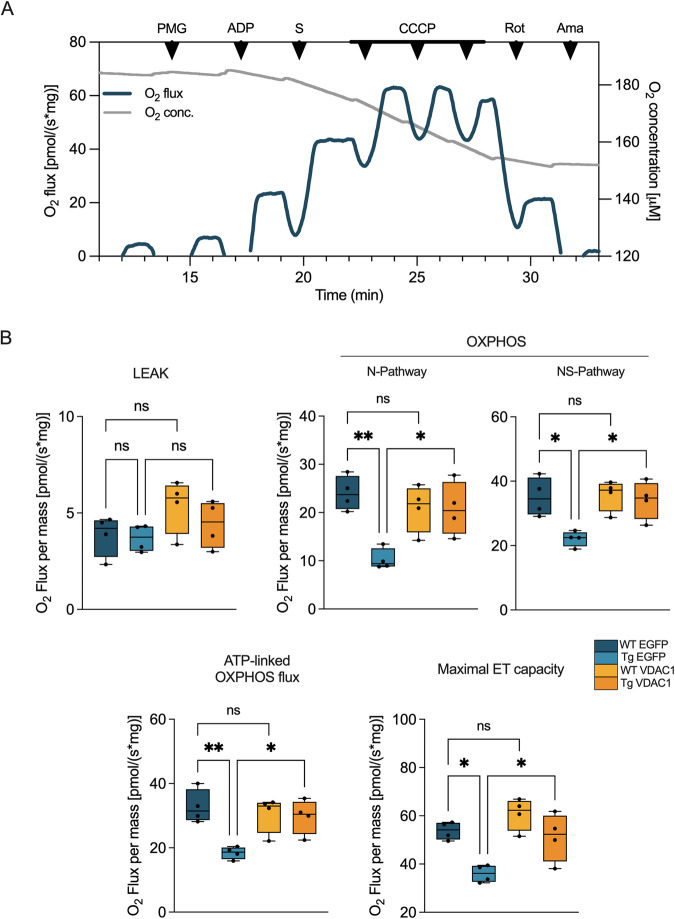
VDAC1 upregulation in transgenic mice restores mitochondrial respiration in spinal cord. **A** Representative trace of oxygen consumption by high-resolution respirometry achieved from fresh spinal cord homogenates of 15-week-old wild-type mice injected with AAV2/5 carrying EGFP. The scheme shows the oxygen consumption and concentration curves along with the SUIT protocol here applied to analyze the main respiratory states. PMG, pyruvate, malate and glutamate; S, succinate; CCCP, carbonyl cyanide 3-chlorophenylhydrazone; Rot, rotenone; Ama, antimycin A. **B** Quantification and comparative analysis of oxygen consumption in wild-type and transgenic mice, either injected with AAV2/5 constructs carrying VDAC1 or EGFP, of spinal cord homogenates relative to the LEAK, N- and NS-pathway (OXPHOS state), ATP-linked OXPHOS respiration and maximal ET capacity. Data are expressed as pmol/s of oxygen per mg of tissue and as median ± SEM of *n* = 4 independent measurements. Data were statistically analyzed by one-way ANOVA, with *p < 0.05 and **p < 0.01; *ns* not significant.

As VDAC1 is the main mitochondrial gateway for metabolites, we investigated the oxygen consumption in fresh spinal cord homogenates by high-resolution respirometry upon the stimulation of the electron transport (ET) chain enzymes with specific substrates. Figure 2A displays a representative curve obtained from wild-type mice expressing EGFP alongside the Substrate-Uncoupler-Inhibitor Titration (SUIT) protocol used here. Briefly, the oxygen consumption corresponding to the non-phosphorylating state (LEAK) was measured without adenylates. Then, the oxidative phosphorylation linked respiration (OXPHOS) driven by NADH-linked substrates, the so-called N-pathway, was measured in the exclusive presence of pyruvate, malate, glutamate and ADP. The further addition of succinate allowed the measurement of the total OXPHOS respiration or NS-pathway. Finally, the maximal electron input to the ET chain (maximal ET capacity) was achieved by titration with the uncoupler carbonyl cyanide 3-chlorophenylhydrazone (CCCP).

In accordance with our previous results, the oxygen flows associated with the main respiratory states, except for the LEAK, were significantly reduced in transgenic mice compared to wild-type both expressing EGFP (Fig. 2B), confirming that ALS phenotype correlates with a general impairment of the respiration in spinal cord motor neurons [19]. However, the overexpression of VDAC1 improved the oxygen consumption of transgenic mice in all the other respiratory states. Precisely, a complete recovery of oxygen fluxes was observed for both N-pathway and NS-pathway (*p* = 0.039 and *p* = 0.037 respectively, *n* = 4), as well as for the OXPHOS flux devoted to ADP phosphorylation (ATP-linked flux, *p* = 0.026, *n* = 4). A similar result was found for the maximal ET capacity (*p* = 0.017, *n* = 4). Interestingly, no significant differences between wild-type mice expressing either EGFP or VDAC1 were detected, indicating that the overexpression of VDAC1 per se does not alter the mitochondrial bioenergetics in healthy spinal cords at this stage (Fig. 2B). Overall, these results suggest that the overexpression of VDAC1 in the spinal cord reliefs mitochondrial respiration in transgenic mice.

### Complex I drives the recovery of respiration in transgenic mice upon VDAC1 upregulation

**Fig. 3:**
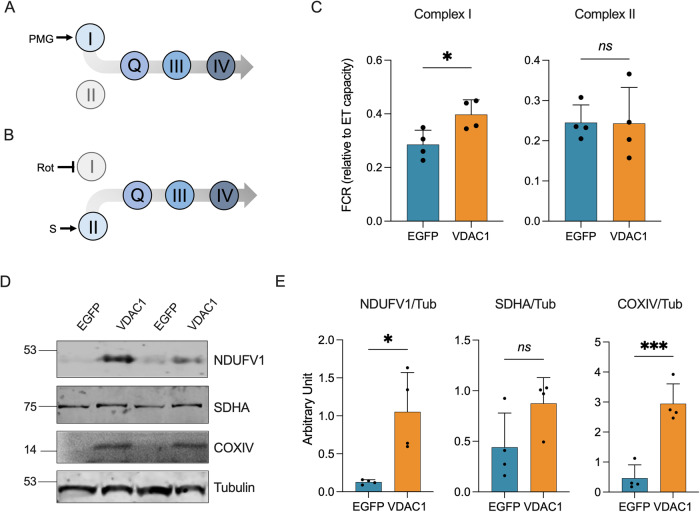
VDAC1 upregulation drives the selective increase of activity and expression of specific ET complexes. **A**, **B** Schematic representation of the experimental set up for the analysis of Complex I and II contribution to the maximal respiration applied in this work. In **A**, the electrons transfer from Complex I to III, thus excluding Complex II, was activated in the presence of ADP and of the NADH-linked substrates exclusively, namely pyruvate (P), malate (M) and glutamate (G). In **B**, activation of the electron transfer from Complex II to III was achieved in the presence of succinate (S) and rotenone (Rot), the last a specific inhibitor of Complex I. **C** Comparative analysis of the specific contribution of Complex I to the total OXPHOS respiration and Complex II to the maximal ET capacity in transgenic mice overexpressing or not VDAC1, calculated as a function of the maximal ET capacity (FCR). **D** Representative Western blots of spinal cord total homogenates extracted from transgenic mice previously injected with VDAC1 or EGFP AAVs showing the levels of the ET chain subunits NDUFV1, SDHA and COXIV. Tubulin was used as loading control. **E** Relative quantification of protein levels of Western blots in (**D**). All the data in the figure are expressed as mean ± SEM of *n* = 4 independent measurements and statistically analyzed by unpaired Student *t*-test, with *p < 0.05 and ***p < 0.001; ns not significant.

We then investigated the relative contributions of each respiratory complex in activating the ET chain. Indeed, the electron transfer to Complex III may occur from the NADH ubiquinone-oxidoreductase (Complex I) or the succinate dehydrogenase (Complex II) via the Q-junction. Our experimental setup allows us to measure the specific contribution of Complex I in the presence of the NADH-linked substrates and ADP but not succinate (Fig. 3A), or Complex II with the supplementation of succinate and also rotenone, a specific inhibitor of Complex I (Fig. 3B**)**. As shown in Fig. 3C, the overexpression of VDAC1 in transgenic mice correlated with a significant increase of Complex I contribution to the respiration, consisting of about a 43% increase in comparison to the genotype-matched mice expressing EGFP (*p* = 0.03, *n* = 4). On the contrary, no significative differences were detected between the groups in terms of Complex II contribution (*p* = 0.96, *n* = 4).

At this point, we queried whether this variation was due to changes in the expression level of specific ET proteins. To this aim, the subunit V1 of NADH ubiquinone-oxidoreductase core (NDUFV1), the subunit A of succinate dehydrogenase (SDHA) and the cytochrome c oxidase (COX IV), also known as Complex IV, were analyzed. In accordance with previous respirometric results, we found that the overexpression of VDAC1 in transgenic mice correlated with a significant increase in the levels of Complex I and IV subunits, but not Complex II subunit (Fig. 3D and Supplementary Fig. 3). Precisely, levels of NDUFAV1 and COX IV were about 7–8 times higher in transgenic mice overexpressing VDAC1 compared to the ones expressing EGFP (*p* = 0.011 and *p* < 0.001 respectively, *n* = 4, Fig. 3E). In contrast, no significant differences were observed in the concentration of SDHA (*p* = 0.086, *n* = 4, Fig. 3E).

Taken together, these results suggest that the observed recovery of mitochondrial respiration is primarily driven by increased activity and expression of Complex I and, in general, the N-pathway.

### VDAC1 upregulation correlates with an increase in the sirtuins level and activity

Within the mitochondria, Complex I generates high levels of NAD^+^ through the reoxidation of NADH. Toward this end, we evaluated the expression and functioning of the main mitochondrial sirtuins, a family of NAD^+^-dependent deacetylases categorized in five classes. Within the mitochondria, Sirt3 and Sirt5 are both involved in the control of energy metabolism and detoxification from ROS [38, 39]. However, while Sirt3 belongs to class I and shows a strong NAD^+^-dependent deacetylase activity, belonging to class III, Sirt5 has mostly demalonylase and desuccinylase activities in presence of NAD^+^, and a weak deacetylase activity [40].

**Fig. 4:**
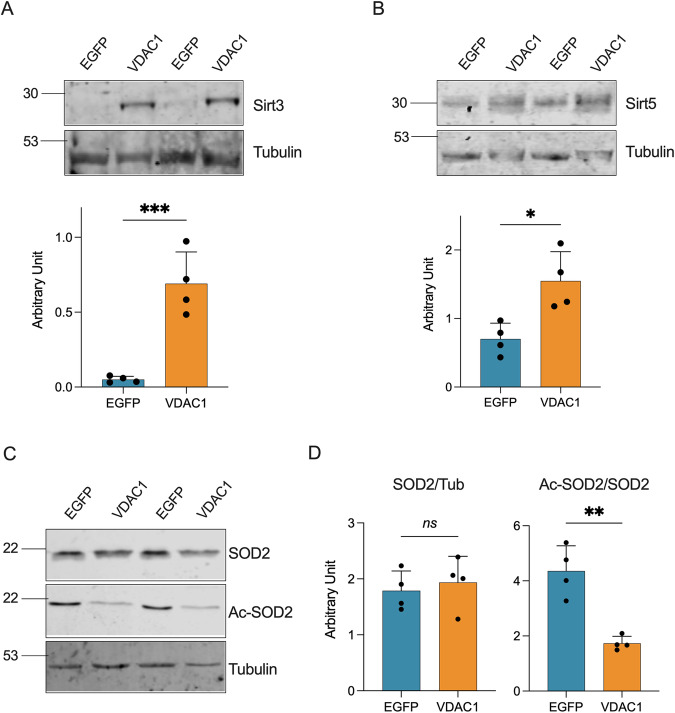
Increase of sirtuins levels and activity correlates with VDAC1 overexpression. **A** Representative Western blots of spinal cord total homogenates extracted from transgenic mice previously injected with VDAC1 or EGFP AAVs showing the levels of Sirt3 and the relative quantification. **B** Representative western blot of spinal cord total homogenates as in **A** showing the levels of Sirt5 and the relative quantification. **C** Representative Western blot of spinal cord total homogenates as in A showing the levels of SOD2 and K68 acetylated SOD2. **D** Relative quantification of protein expression of Western blot in (**C**). In all the blots, Tubulin was used as loading control. All the data are expressed as mean ± SEM of *n* = 4 independent measurements and statistically analyzed by unpaired Student *t*-test, with **p < 0.01 and ***p < 0.001; *ns* not significant.

As shown in Fig. 4A and Supplementary Fig. 4, the overexpression of VDAC1 correlated with more than 10 times increase of Sirt3 in transgenic mice (*p* < 0.001, *n* = 4). Although less marked, we noticed an increase in Sirt5 expression upon VDAC1 upregulation (*p* = 0.013, *n* = 4, Fig. 4B and Supplementary Fig. 5).

Among other things, Sirt3 positively regulates the activity of MnSOD (SOD2) by the deacetylation of the lysine residues 68 and 122 [41]. Thus, the levels of total and acetylated SOD2 were used here as a marker of Sirt3 activity. As showed in Fig. 4C, D and Supplementary Fig. 6, while the total level of SOD2 remained unchanged between the two groups (*p* = 0.636, *n* = 4), the level of K68 acetylated SOD2 was significantly reduced upon VDAC1 upregulation (*p* = 0.0015, *n* = 4). Overall, these data suggest that an increased expression of the main porin isoform has a positive effect on the main mitochondrial sirtuins expression and on the activity of Sirt3.

### VDAC1 enhances Tom20 expression

**Fig. 5:**
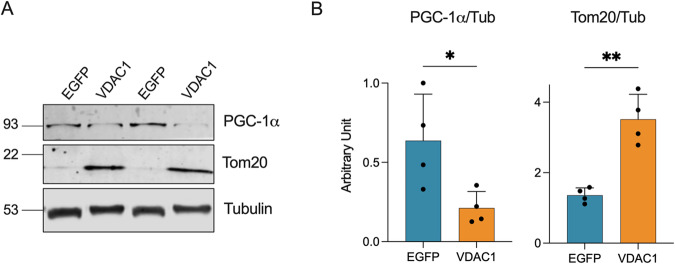
Downregulation of PGC-1α inversely correlates with Tom20 expression in transgenic mice injected with VDAC1. **A** Representative Western blots of spinal cord total homogenates extracted from transgenic mice previously injected with VDAC1 or EGFP AAVs showing the levels of PGC-1α and Tom20. Tubulin was used as loading control. **B** Relative quantification of protein expression of Western blot in (**A**). Data are expressed as mean ± SEM of *n* = 4 independent measurements and statistically analyzed by unpaired Student *t*-test, with *p < 0.05 and **p < 0.01.

To understand the molecular mechanisms behind the process driven by VDAC1 overexpression, we investigated whether the selective increase of specific mitochondrial proteins was due to the activation of mitochondrial biogenesis or some other mitochondrial quality control (MQC) mechanisms. To this aim, we analyzed the expression level of the main transcriptional coactivator of mitochondrial biogenesis, the Peroxisome proliferator-activated receptor-gamma coactivator-1 alpha (PGC-1α), and the receptor subunit of the TOM complex, Tom20. The TOM complex is known to regulate the entry of mitochondrial protein precursors from the cytosol into the organelle and to participate in MQC pathways [42]. As reported in Fig. 5A and Supplementary Fig. 7, upon VDAC1 overexpression, we observed a slight but significant reduction of PGC-1α levels (*p* = 0.034, *n* = 4, Fig. 5B), which would suggest a lack of induction of biogenesis. Instead, overexpression of VDAC1 correlated with a robust increase in Tom20 expression (*p* = 0.0011, *n* = 4, Fig. 5B). Taken together, these data suggest an involvement of the TOM complex in the VDAC1-mediated increased expression of certain mitochondrial proteins. This is not surprising as the import of a subset of mitochondrial protein precursors can be positively or negatively regulated in the cytosol by precursor-specific reactions [43].

## Discussion

The alteration of the proper VDAC1 functioning in ALS is detrimental to mitochondrial homeostasis. Indeed, the interaction of misfolded proteins with VDAC1 decreases the metabolic exchanges across the OMM, reducing mitochondrial ADP entry and, thus, ADP phosphorylation [33, 44]. However, VDAC1 is much more than a mere channel for ions and metabolites in and out of mitochondria. Its physiological interaction with HKs allows it to combine glycolysis with oxidative phosphorylation, contributing to the maintenance of proper ATP/ADP levels and the inhibition of apoptosis [28, 45, 46]. In contrast, the inactivation of VDAC1 gene is enough to cause the collapse of the mitochondrial respiratory system, prompting the cell towards a metabolic rewiring to overcome the mitochondrial failure [47–49]. Therefore, VDAC1 behaves as a molecular switch that, if turned off, may have serious consequences for the cell survival. It could behave as a ‘sensor’ of mitochondrial metabolism and, as such, activate survival and/or mitochondrial quality control signals. Not coincidentally, VDAC3 isoform has been recently shown to function as a sensor of the oxidative state [50, 51].

**Fig. 6:**
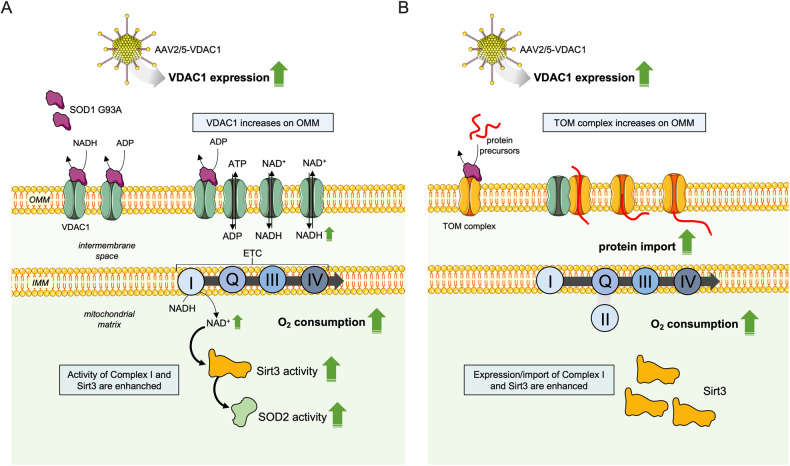
Proposed model on the role of VDAC1 upregulation in recover the mitochondrial dysfunction in ALS transgenic mice. **A**, **B** The overexpression of VDAC1 in SOD1 G93A transgenic mice results in a general restore of the mitochondrial respiratory profile that possibly depends on two strictly interconnected mechanisms. The exogenous expression of VDAC1 mediated by AAV2/5 vector partially compensates for endogenous VDAC1 molecules whose functioning is compromised by the toxic interaction with SOD1 mutants. This promotes an increase of VDAC1 in the OMM and of the metabolic exchanges across the OMM, especially of ADP/ATP and NAD^+^/NADH. In particular, NADH feeds Complex I stimulating the activity of the N-pathway. At the same time, the reoxidation of NADH to NAD^+^ by Complex I stimulates the activity of Sirt3, a general regulator of the whole mitochondrial and ET chain activity (**A**). VDAC1 upregulation correlates with an increase in the levels of Tom20, a key component of TOM complex, that promotes the increase of mitochondrial proteins import within the organelle, as for Complex I and Sirt3 (**B**). The figure was partly generated using Servier Medical Art, provided by Servier, licensed under a Creative Commons Attribution 3.0 unported license.

To ameliorate the compromised mitochondrial functionality in ALS, we therefore explored the inedited strategy to upregulate VDAC1 expression in the spinal cord affected by the disease. This was achieved by delivering the correspondent gene by a recombinant AAV2/5 vector to the spinal cord of SOD1 G93A transgenic mice, a widely used and well-characterized mouse model of ALS [52]. Herein, we showed that a robust, stable and long-term overexpression of VDAC1 in symptomatic mice, promoted the complete recovery of the whole oxygen consumption profile to the levels of wild-type mice. This result strongly suggests that VDAC1 improves the mitochondrial bioenergetic in spinal cord of transgenic mice. In particular, the beneficial effect appears to be the result of two interconnected mechanisms. As schematized in Fig. 6, the upregulation of VDAC1 enhances the metabolic exchanges through the OMM by stimulating: (i) the functioning and activity of mitochondrial enzymes Complex I and sirtuins, and (ii) the expression of the same mitochondrial proteins.

The inhibition of Complex I characterizes both familial and sporadic ALS [53–55] and, among other things, it also depends on the limited availability of NADH due to a reduced import/export of nicotinamide cofactors via VDAC1. In our respirometric experiments, the recovery of respiration was already noticeable when the ET chain was stimulated with the exclusive presence of NADH-linked substrates; accordingly, the contribution of Complex I to the maximal ET capacity, but not Complex II, was significantly increased. The same was observed for the expression of Complex I subunit NDUFV1. Therefore, these data suggest that a more efficient exchange of NAD^+^/NADH due to VDAC1 upregulation increases NADH availability and utilization due to an increase in the levels of Complex I (Fig. 6A). At the same time, the NAD^+^ derived from NADH reoxidation behaves as a potent activator of sirtuins, and in particular of Sirt3 [56–58]. Besides SOD2, Sirt3 regulates many other mitochondrial enzymes, including several ET subunits as NDUFA9 of Complex I by deacetylation [59]. Conversely, high-level of acetylation of mitochondrial proteins, as found in patient-derived iPSCs carrying different ALS mutations, is a direct consequence of the reduced activity of Sirt3 and correlates with a significant inhibition of mitochondrial respiration [60]. In our work, the recovery of respiration correlates with an increase of Sirt3 activity, here tested by analyzing the acetylation level of its downstream target SOD2 (Fig. 6A). In parallel, we observed a significant increase in the levels of Sirt3 and Sirt5, as previously found for Complex I. Interestingly, in an earlier work we already shown that Sirt3 expression in transgenic mice of the same genetic background is downregulated compared to wild-type [19].

The selective increase of mitochondrial proteins NDUFV1, Sirt3 and Sirt5, but not SDHA or SOD2, seems to be independent from the occurrence of mitochondrial biogenesis, as suggested by the reduced levels of PGC-1α detected in transgenic mice injected with VDAC1. Conversely, we noticed a significant increase in the levels of the TOM complex marker Tom20. TOM complex represents the primary entry gate for more than 90% of mitochondrial proteins, with different subunits of this complex playing distinct roles in pre-protein recognition and import [61]. At first glance, the reduction in the expression levels of PGC-1α could appear in contrast with the recovery of the mitochondrial functions previously described. As a matter of fact, PGC-1α is a transcriptional co-activator widely considered the main modulator of the mitochondrial biogenesis and, more generally, of the organelle homeostasis, being involved in the maintenance of the proper respiratory capacity activity and the regulation of the OXPHOS proteins expression [62]. In contrast with this, the genetic inactivation of the gene encoding PGC-1α, as occurs in knockout models, led to a reduction of the OXPHOS protein content of only 10–50%, suggesting that the downstream transcription factors are however mostly active even in the absence of PGC-1α [63]. Furthermore, an inverse correlation between the expression of PGC-1α and TOM complex (specifically, the subunit Tom22) was already noticed in aged human skeletal muscle, and was interpreted as an attempt to compensate the reduced mitochondrial biogenesis with an increase of mitochondrial proteins import and assembly within the organelle [64]. Therefore, we can speculate that, in our model, a similar process could occur and strictly depends on the upregulation of VDAC1.

In addition to its metabolic role, VDAC1 has recently been associated with mitochondrial protein biogenesis with a dual role [65]. The porin promotes both the import of inner mitochondrial membrane carrier proteins as well as the assembly and stability of the TOM complex, by modulating the integration of the central receptor Tom22 into the mature translocase [66–68]. Moreover, function and integrity of mitochondria depend on the activity of different MQC mechanisms, such as the restoration of mitochondrial protein import, respiratory capacity and mitochondrial proteostasis, that involve, directly or indirectly, the TOM complex. In pathological contexts, MQC pathways can play a protective role or, if malfunctioning, negatively affect disease progression [69]. Although other studies are necessary, we cannot exclude that in transgenic ALS mice the overexpression of VDAC1 drives the activation of one of these MQC pathways, improving the import of specific mitochondrial proteins via the TOM complex (Fig. 6B).

Interestingly, it was recently reported that Sirt3 regulates the level of HIF-1α in astrocytes [70], an important transcription factor that along with mitochondrial and cell survival also controls the expression of the VDAC1 gene [71]. Under reduced oxygen levels, Sirt3 stabilizes a HIF-1α/Tom22 circuit that leads to the expression of Tom20, thereby reactivating mitochondrial functions [70]. Therefore, also considering these literature data, we can hypothesize that in ALS VDAC1 promotes an adaptation signal to disease-induced stress by stabilizing the Complex I-Sirt3 axis, involving also the TOM complex.

In conclusion, the data shown here highlight the effective use in ALS of recombinant AAV vectors for intraspinal transgene delivery and point to the upregulation of VDAC1 as a possible therapeutic molecule to restore proper mitochondrial functioning in the ALS-affected tissues.

## Materials and methods

### Mice

The transgenic mice B6.Cg-Tg(SOD1*G93A)1Gur/J (JAX ref. no. 004435), expressing the human SOD1 G93A mutant in high copy number, and C57BL6/J wild-type mice were purchased from The Jackson Laboratory (Bar Harbor, ME, USA). To obtain transgenic and wild-type mice, the colony was maintained by breeding male hemizygous carriers to wild-type females. 8 to 10 mice for each experimental group, with an approximately equal number of males and females, at the age-matched of 15-week-old were used in this study. Mice were housed 4-5 per cage, kept on 12 h light/dark cycle and were given *ad libitum* access to food and water. Mice were weighed weekly starting from week 4. Body weight increase was calculated as the difference between the maximum weight reached at the final point and the weight at week 4.

All the experimental procedures were carried out according to the Italian Guidelines for Animal Care (D.L. 116/92 and 26/2014) and in compliance with the European Communities Council Directives (2010/63/EU). All protocols were approved by the Ethical Committee for animal experimentation at the University of Catania (OPBA, project ref. no. 334). All measures were adequately taken to minimize the number of animals.

### Genotyping

Transgenic mice were identified by PCR after the extraction of genomic DNA from small tail biopsies. According to manufacturer protocol, primers 5′-CAT CAG CCC TAA TCC ATC TGA-3′ and 5′-CGC GAC TAA CAA TCA AAG TGA-3′ were employed to amplify a 236 bp product from exon 4 of the human SOD1 gene within the transgene construct. The following primers 5′-CTA GGC CAC AGA ATT GAA AGA TCT-3′ and 5′-GTA GGT GGA AAT TCT AGC ATC ATC C-3′ were used to amplify a 324 bp product of the positive/internal control corresponding to the endogenous interleukin 2 gene.

### AAV constructs preparation and delivery

The AAV vectors carrying the encoding sequence of the EGFP gene or VDAC1-2A-EGFP, both under the control of the CAG promoter, were generated and purchased by Vector Biolabs (Malvern, PA, USA). To achieve a widespread expression of target genes in the spinal cord, AAV constructs were packaged into AAV2/5 serotype and directly injected at the lumbar spinal cord site of neonatal pups at P2, as previously performed by Ayers and colleagues [37]. Briefly, pups were cryoanesthetized on ice for about 3 min or up to stop all the movements. Then, 2 μl of the virus was slowly injected into the spinal cord using a 10 μl Hamilton syringe (1″ needle, 30° bevel). Needle insertion into the vertebral column started parallel to the back and at about 5 mm from the tail base, then angled to 50–60° [37]. After the injection, the pups were placed on a warming blanket and then returned to their home cage.

### Spinal cord extraction and conservation

Animals were sacrificed at week 15 by CO_2_ asphyxiation and spinal cords were rapidly removed. For respirometric analysis, tissues were kept in ice-cold BIOPS buffer (10 mM Ca EGTA, 20 mM imidazole, 20 mM taurine, 50 mM K-MES, 0.5 mM DTT, 6.56 mM MgCl2, 5.77 mM ATP, 15 mM phosphocreatine, all purchased from Sigma-Aldrich, St. Luis, MO, USA) to preserve the mitochondrial functions and integrity [72]. For Western blot analysis, spinal cords were frozen in dry ice and stored at –80 °C.

### High-resolution respirometry experiments

Mitochondrial oxygen consumption was assayed in fresh spinal cord homogenates by high-resolution respirometry, using the double-chamber system O2k FluoRespirometer (Oroboros Instruments, Innsbruck, Austria). Fresh spinal cord homogenates were prepared within 1 h from the extraction by using a Potter Elvehjem tissue homogenizer in mitochondrial respiration buffer Mir06 (Oroboros Instruments) and immediately used for respirometric analysis. Tissue homogenization protocol was tested for mitochondrial membrane integrity by cytochrome c (cyt c) assay [19, 73]. For each analysis, a volume of homogenate equivalent to 2 mg of the original tissue was added to the cuvette.

Instrumental and chemical background fluxes were calibrated as a function of the oxygen concentration using DatLab software (version 7.4.0.1, Oroboros Instruments). The rate of oxygen consumption relative to each respiratory state was expressed as pmol/s per milligram of tissue. The contribution of Complex I to the OXPHOS respiration and of Complex II to the maximal ET capacity was calculated as Flux Control Ratio (FCR) by normalizing the oxygen consumption correspondent to N- and S-pathway for the maximal ET capacity [74–76]. The oxygen flux relative to ATP production was calculated as the difference between total OXPHOS and the LEAK respiration [77]. All the experiments were performed in Mir06 (Oroboros Instrument) at 37 °C under constant stirring at 750 rpm. A set of animals *n* = 4 per experimental group was employed.

### SUIT protocol

A SUIT protocol aimed at investigating the main respiratory states was performed as previously described [19, 78]. Briefly, the LEAK state was assayed after the closure of the cuvette and the addition of 10 mM pyruvate, 2 mM malate and 10 mM glutamate. The subsequent addition of saturating concentration of ADP (5 mM) allowed us to measure the N-pathway, i.e., the OXPHOS respiration exclusively driven by NADH-linked substrates. Further supplementation with 10 mM succinate allowed to achieve the total OXPHOS respiration or NS-pathway. Then, a titration with 0.5 μM of the uncoupler CCCP was performed up to reach the maximal ET capacity. Finally, ET complexes I and III were selectively inhibited by the addition of 2 μM rotenone and 2.5 μM antimycin A, respectively, to obtain the residual oxygen consumption (ROX). All chemicals were purchased from Sigma-Aldrich.

### Western blot analysis

Frozen spinal cords were homogenized in T-PER buffer (ThermoFisher, Waltham, MA, USA) supplemented with cOmplete Protease Inhibitor Cocktail (Roche, Basel, Switzerland) and phosphatase inhibitor (Sigma-Aldrich). Total protein lysates were quantified by Pierce BCA Protein Assay Kit (ThermoFisher) and added to NuPAGE LDS sample buffer, supplemented with Sample reducing agent (ThermoFisher). Proteins were separated on NuPAGE Bis-Tris polyacrylamide gels (ThermoFisher) at 150 V for 50 min and transferred to nitrocellulose membranes (GE Healthcare, Boston, MA, USA) using the semi-dry system PerfectBlue Electro Blotter (Peqlab, Erlangen, Germany). Membranes were blocked in 5% BSA or non-fat milk in PBS with 0.1% Tween-20. Full or portions of membranes were incubated overnight at 4 °C with the following primary antibodies against EGFP (Cell Signaling, Danvers, MA, USA, ref. no. 2956S, 1:1000), VDAC1 (Abcam, Cambridge, UK, ref. no. ab14734, 1:1000), NDUFV1 (Immunological Sciences, ref. no. AB-83826, 1:500), SDHA (Abcam, ref. no. ab137040, 1:500), COX IV (Cell Signaling, ref. no. 4850, 1:1000), Sirt3 (Immunological Sciences, ref. no. AB-84353, 1:500), Sirt5 (Abcam, ref. no. ab105040, 1:1000), SOD2/MnSOD (Abcam, ref. no. ab13533, 1:500), SOD2/MnSOD acetyl K68 (Abcam, ref. no. ab137037, 1:500), PGC-1α (Abcam, ref. no. ab188102, 1:500), Tom20 (Abcam, ref. no. ab186735, 1:500), Tubulin (Cell Signaling, ref. no. 2146, 1:1000). Then, membranes were incubated with IRDye conjugated secondary antibodies (LI-COR Biosciences, Lincoln, NE, USA, 1:25.000). Signals were detected using Odyssey Imaging System (LI-COR Biosciences). Band quantification was performed by densitometric analysis using Image Studio Lite software (LI-COR Biosciences). A set of animals *n* = 4 per experimental group was employed.

### Statistical analysis

Data were expressed as a median or mean ± SEM and statistically analyzed by one-way ANOVA or unpaired Student *t*-test using GraphPad Prism version 9 (GraphPad Software, Boston, MA, USA). The values of *p* < 0.05, *p* < 0.01 and *p* < 0.001 were taken as significant.

### Supplementary information

Supplementary Material

Original data

## Data Availability

All data generated or analyzed during this study are included in this article and in Supplementary files.
