# Supplementary material for: AAV-mediated upregulation of VDAC1 rescues the mitochondrial respiration and sirtuins expression in a SOD1 mouse model of inherited ALS
Source: Cell Death Discov. 2024 Apr 16;10:178. doi: 10.1038/s41420-024-01949-w (PMC11021507; doi:10.1038/s41420-024-01949-w)
Supplement: Supplementary file 1 — Supplementary Material [file 41420_2024_1949_MOESM1_ESM.docx]

**Supplementary Materials for**

**AAV-mediated upregulation of VDAC1 rescues the mitochondrial respiration and sirtuins expression in a SOD1 mouse model of inherited ALS**

Andrea Magrì^1,2*^, Cristiana Lucia Rita Lipari^3*^, Antonella Caccamo^4^, Giuseppe Battiato^3^,

Stefano Conti Nibali^3^, Vito De Pinto^2,3^, Francesca Guarino^2,3^, Angela Messina^1,2#^

1. Department of Biological, Geological and Environmental Sciences, University of Catania, Via S. Sofia 97, 95123 Catania, Italy
2. we.MitoBiotech s.r.l., C.so Italia 172, 95125, Catania, Italy
3. Department of Biomedical and Biotechnological Sciences, University of Catania, Via S. Sofia 97, 95123 Catania, Italy
4. Department of Chemical, Biological, Pharmaceutical and Environmental Sciences, University of Messina, V.le F. Stagno d’Alcontres 32, 98166 Messina, Italy

* These authors contributed equally to this work

# Author to whom correspondence should be addressed:

Angela Messina

Department of Biological, Geological and Environmental Sciences

University of Catania

Via S. Sofia 97, 95123 Catania, Italy

Tel: +39 095 7384231

Email: mess@unict.it

**Supplementary Figure 1** Analysis of EGFP expression by Western blot in total homogenates of spinal cord from n=4 wild-type mice previously injected with the AAV2/5 vector carrying the EGFP sequence. Mice were sacrificed after 15 weeks from the injection. Tubulin was used as loading control.

**Supplementary Figure 2** Full scans of the Western blot relative to Figure 1C showing all the replicates used for quantification. The box in red indicates the portion displayed in the correspondent main figure.

**Supplementary Figure 3** Full scans of the Western blot relative to Figure 3D showing all the replicates used for quantification. The box in red indicates the portion displayed in the correspondent main figure.

**Supplementary Figure 4** Full scans of the Western blot relative to Figure 4A showing all the replicates used for quantification. The box in red indicates the portion displayed in the correspondent main figure.

**Supplementary Figure 5** Full scan of the Western blot relative to Figure 4B showing all the replicates used for quantification. The box in red indicates the portion displayed in the correspondent main figure.

**Supplementary Figure 6** Full scans of the Western blot relative to Figure 4C showing all the replicates used for quantification. The box in red indicates the portion displayed in the correspondent main figure.

**Supplementary Figure 7** Full scans of the Western blot relative to Figure 5A showing all the replicates used for quantification. The box in red indicates the portion displayed in the correspondent main figure.
