# Supplementary figures and images for: AAV-mediated upregulation of VDAC1 rescues the mitochondrial respiration and sirtuins expression in a SOD1 mouse model of inherited ALS
Source: Cell Death Discov. 2024 Apr 16;10:178. doi: 10.1038/s41420-024-01949-w (PMC11021507; doi:10.1038/s41420-024-01949-w)

Figure 1C

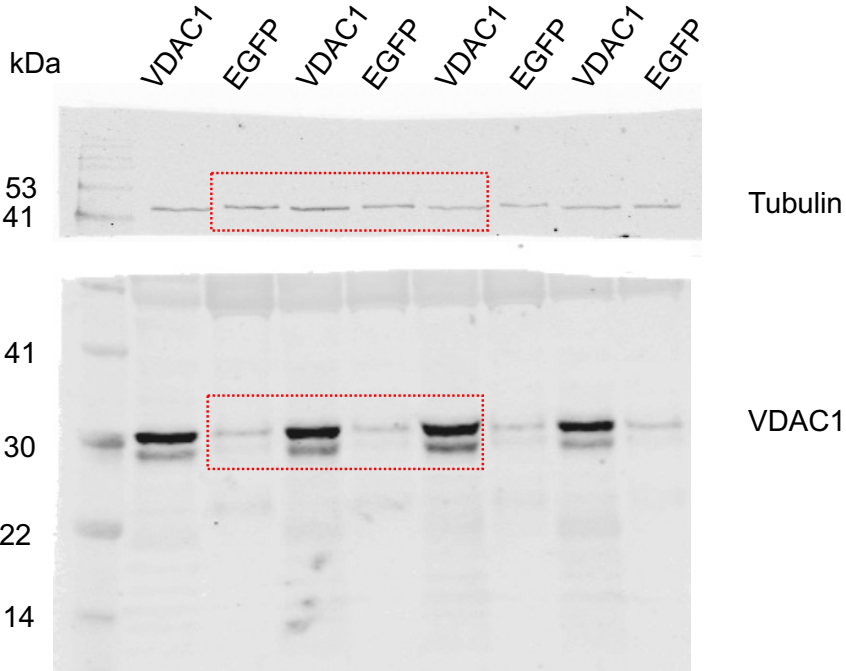

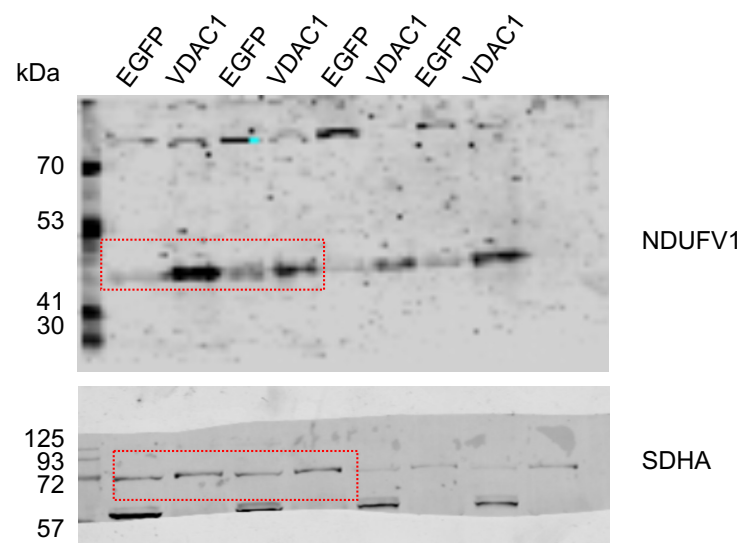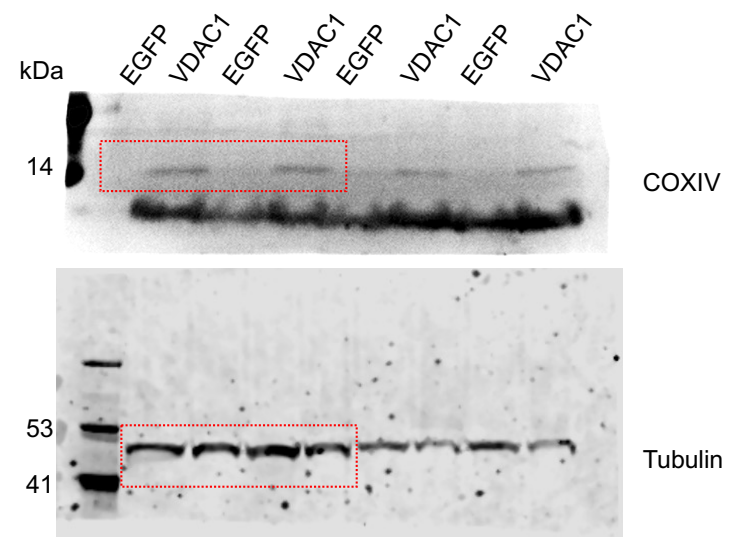

Figure 4A

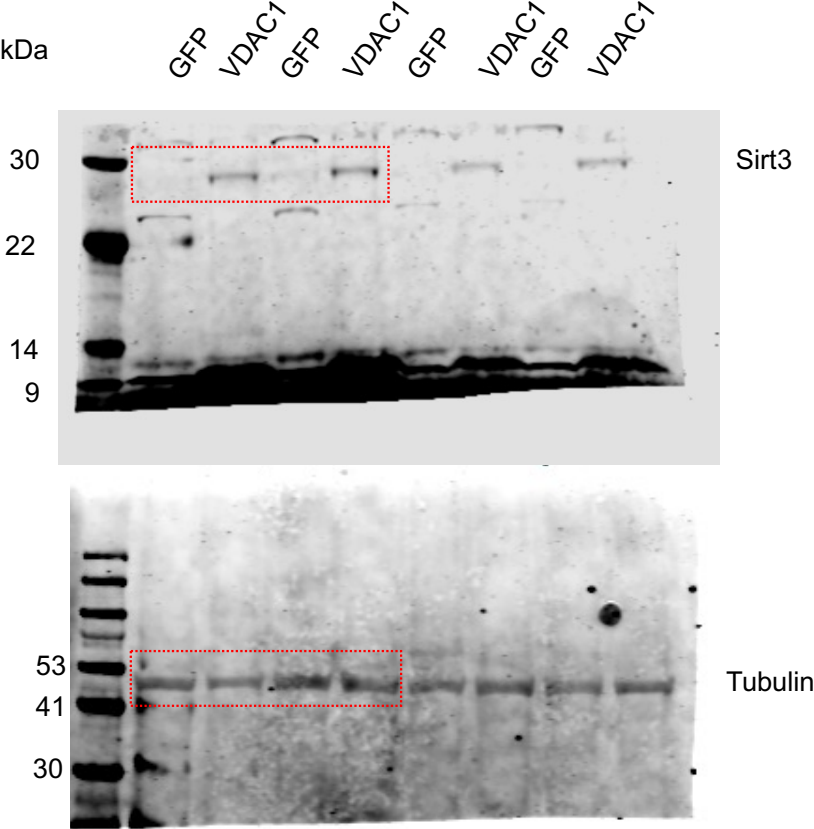

Figure 4B

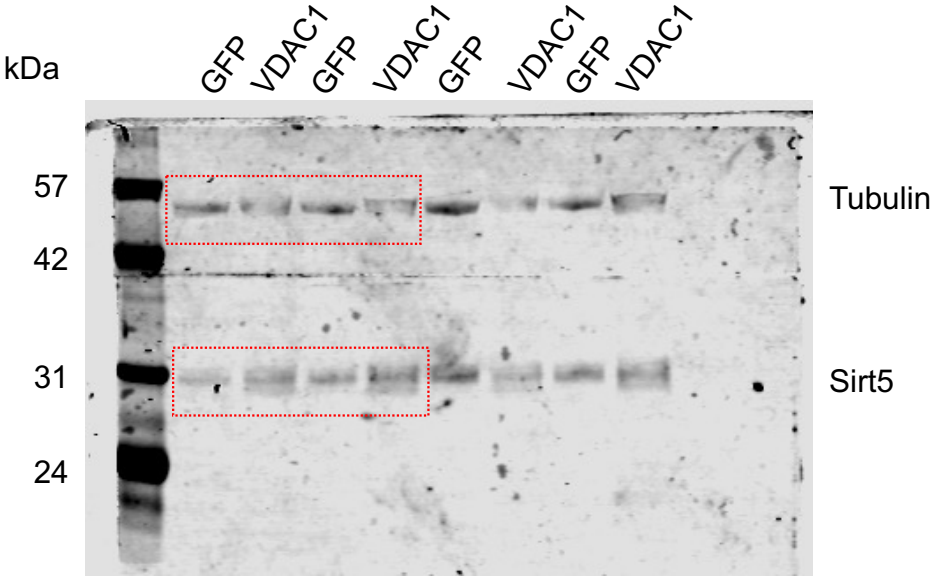

Figure 4C

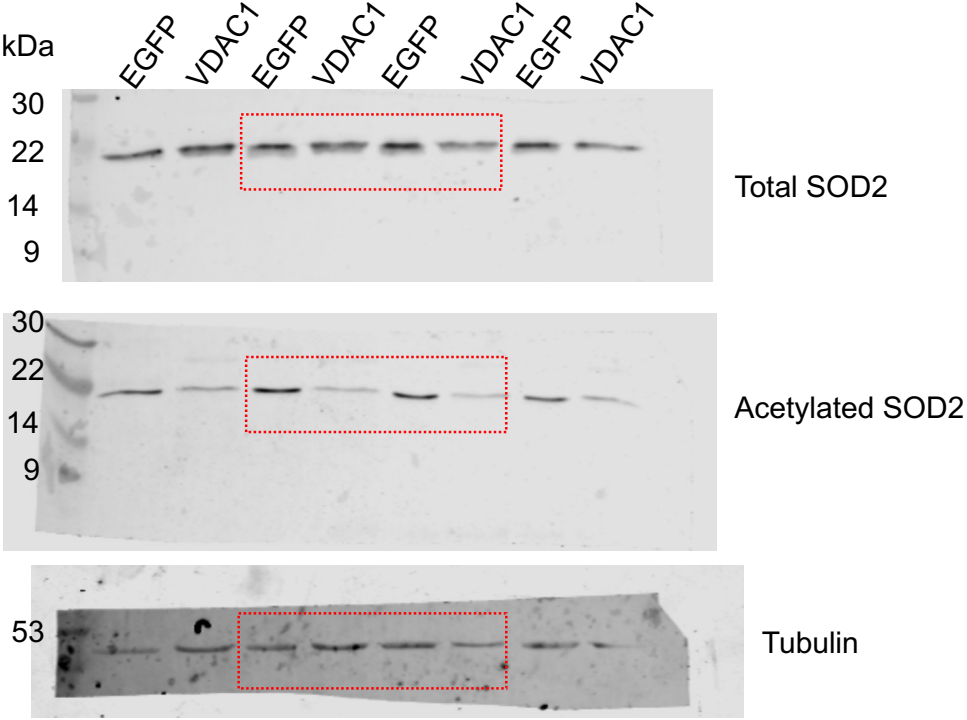

### Figure 5A

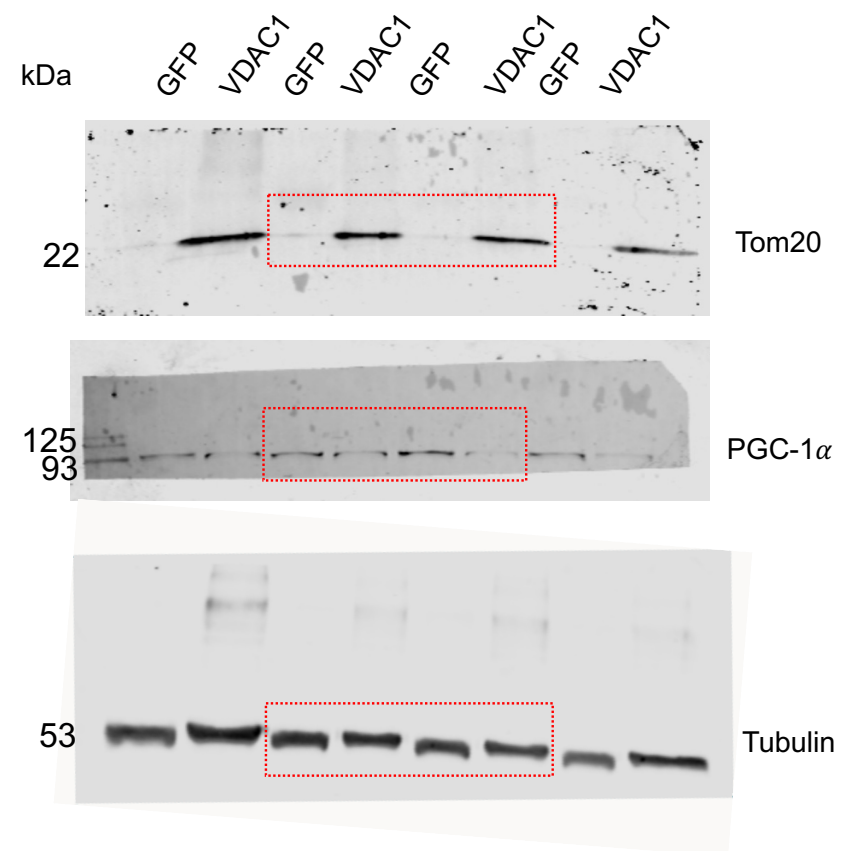

Supplement: Supplementary file 2 — Original data [file 41420_2024_1949_MOESM2_ESM.pdf]
